# Tunisian’s largest Psychiatric emergency department in the context of the COVID-19 lockdown

**DOI:** 10.1192/j.eurpsy.2022.1502

**Published:** 2022-09-01

**Authors:** N. Sayari, A. Maamri, O. Charaa, A. Hajri, H. Zalila

**Affiliations:** Razi Hospital, Emergency And Outpatient Departement, manouba, Tunisia

**Keywords:** emergency, lockdown, Covid-19, confinement

## Abstract

**Introduction:**

General Lockdown was first declared in Tunisia from March 20^th^ to May 4^th^ 2020 to contain the spread of COVID19 pandemic, the last sanitary lockdown period was declared from July 12^th^ to august 1^st^ in 2021. Psychiatric emergency access and consultation has been affected by the confinement. RAZI Hospital Emergency Department (RHED) is the only emergency department in Tunisia specialized in psychiatry. Thus making it the most representative psychiatric emergency health care service in Tunisia.

**Objectives:**

To assess changes in patients flow and admission rates in RHED in the context of the COVID-19 lockdown

**Methods:**

We examined emergency room records and the hospital’s computer admission database during the first and the last COVID19 lockdowns and compared it to the same period of the previous year.

**Results:**

The number of consultations was significantly lower in 2020 lockdown (N = 577) compared the same period in 2019 (N = 1525) (p<10^−3^). We observed a drop in RHED emergency hospitalization rate from 45.57% to 29.81% during this study period. The number of consultations per day was significantly lower during the first lockdown (N= 12.44) compared to the last lockdown (N=26.61) (*p*<10^−3^), the hospitalization rate rose from 29.81% during the first lockdown to 44.36% during the last.

**Conclusions:**

Fear of COVID19 contamination and lockdown limitation had a huge impact on RHED visits and admissions. Medical team had to adjust in order to prevent further delay in acute psychiatric care.

**Disclosure:**

No significant relationships.

